# Fish and Crayfish Toxicology

**DOI:** 10.1155/2014/768689

**Published:** 2014-05-11

**Authors:** Josef Velíšek, Zdenka Svobodova, Antonín Kouba, Zhi-Hua Li

**Affiliations:** ^1^Research Institute of Fish Culture and Hydrobiology, Faculty of Fisheries and Protection of Waters, South Bohemian Research Center of Aquaculture and Biodiversity of Hydrocenoses, University of South Bohemia in Ceske Budejovice, Zatisi 728/II, 389 25 Vodnany, Czech Republic; ^2^Faculty of Veterinary Hygiene and Ecology, University of Veterinary and Pharmaceutical Sciences Brno, Palackého 1/3, 612 42 Brno, Czech Republic; ^3^Yangtze River Fisheries Research Institute, Chinese Academy of Fishery Sciences, No. 8, 1st Wudayuan Road, East Lake Hi-Tech Development Zone, Wuhan, Hubei 430223, China

Pollution of the environment and its protection have become increasingly to the forefront of mankind's concerns. Aquatic ecosystems are exposed to a continual inflow of pollutants of both natural and anthropogenic origin. These substances can in many cases result in negative changes in water quality. Water-inhabiting organisms constitute one of the essential components of the ecosystem. Fish and crayfish are a very important part of the aquatic ecosystem and simultaneously are also important economic organisms for human consumption. Over the last 50 years, there have been significant developments in the field of aquatic toxicology. The subject of aquatic toxicology is research and estimation of the effect of xenobiotic on aquatic ecosystem and organisms living there. This issue on fish and crayfish toxicology represents recent results of several studies, mostly carried out on fish. Laboratories from United States, China, and Czech Republic submitted 16 papers. Altogether, 7 original papers and 1 review were accepted after the reviewing process. The following briefly summarizes highlights of the published papers.

*Phytoestrogens.* Two papers with emphasis on effects of phytoestrogens on fish have been accepted. In the first paper, A. C. Brown et al. evaluated effects of phytoestrogens produced by plants on female Siamese fighting fish (*Betta splendens*). In this study, authors found no effects of phytoestrogens in natural concentration (1 *μ*g L^−1^) of *β*-sitosterol and genistein on steroids and gonads. Effect of phytoestrogens on behavior was only detected in higher concentrations of 1000 *μ*g L^−1^. In the second study, E. D. Clotfelter and H. K. Gendelman evaluated effects of phytoestrogens on male reproductive function of Siamese fighting fish. Their observation suggests that acute exposure to waterborne phytoestrogens during activation does not reduce the motility of fish sperm.

*Effects of Xenobiotics on Fish.* Three papers with emphasis on effects of xenobiotic on fish have been accepted. DEET (N,N-diethyl-m-toluamide), the most common active ingredient in insect repellents, as described by A. Slaninova et al., was shown to affect the hematological, biochemical, and oxidative stress parameters in common carp (*Cyprinus carpio* L.) at a concentration of 1 mg L^−1^. A study by M. Bartoskova et al. investigated the effects of fluoroquinolone norfloxacin (antibacterial agents) on selected oxidative stress parameters in zebrafish (*Danio rerio*). From their results, we can conclude that norfloxacin has a negative impact on specific biochemical processes connected with the production of reactive oxygen species in tested fish. The third study by V. Stancova et al. examined the long term effect of a mixture of ibuprofen, diclofenac, and carbamazepine on early life stages of tench (*Tinca tinca*). Exposure to the mixture of pharmaceuticals at a concentration of 60 *μ*g L^−1^ for each substance caused an increase in mortality as well as increase in growth, elevated incidence of malformations, and histopathological changes of liver, kidney, skin, and gill. However, environmentally relevant concentrations (0.02 and 0.2 *μ*g L^−1^) used in this experiment did not result in toxic impairment of early life stage of tench.

*Mycotoxins.* One important paper with emphasis on effects of deoxynivalenol (DON), produced by the* Fusarium *genus, a major contaminant of cereal grains, on rainbow trout (*Oncorhynchus mykiss*), was accepted. DON in a dose of 2 mg kg^−1^ feed, as described by I. Matejova et al., was shown to affect hematological, biochemical, and histopathological parameters in this fish.

*Fish Nutrition.* Y. Zhu et al. conducted experiments for the detection of effects of different diets on the growth performance and hypoxia adaptation capacity of Mississippi paddlefish (*Polyodon spathula*) larvae. The larval paddlefish fed with an appropriate proportion of live food and formulated diets exhibited improved adaptive capacity to hypoxia.

*Fish as Model Organisms.* One review by S. Dong et al. dealt with attempts to find the most suitable environmentally relevant fish model for toxicity testing. This review provides a literature survey highlighting the steady increase of ecotoxicological research on marine medaka (*Oryzias melastigma*), summarizes the advantages of using marine medaka as a tool for toxicological research, and promotes the utilization of this fish in future studies.

We hope that this issue will attract interest from many people, including scientists, university teachers, and students studying aquatic toxicology.

